# Characteristics of the First Italian Older Adults Vaccinated with an Adjuvanted Respiratory Syncytial Virus (RSV) Vaccine

**DOI:** 10.3390/medicina61010067

**Published:** 2025-01-03

**Authors:** Alexander Domnich, Andrea Orsi, Piero Luigi Lai, Elvira Massaro, Carlo-Simone Trombetta, Julieta Pastorino, Charlott Roihl, Marianna Pianta, Giancarlo Icardi, Donatella Panatto

**Affiliations:** 1 Hygiene Unit, San Martino Policlinico Hospital, IRCCS for Oncology and Neurosciences, 16132 Genoa, Italy; andrea.orsi@unige.it (A.O.); icardi@unige.it (G.I.); 2 Department of Health Sciences, University of Genoa, 16132 Genoa, Italy; piero.luigi.lai@unige.it (P.L.L.); elvira.massaro@edu.unige.it (E.M.); 5285039@studenti.unige.it (C.-S.T.); 6556101@studenti.unige.it (J.P.); 5731134@studenti.unige.it (C.R.); 4419417@studenti.unige.it (M.P.); panatto@unige.it (D.P.); 3 Interuniversity Research Centre on Influenza and Other Transmissible Infections (CIRI-IT), 16132 Genoa, Italy

**Keywords:** respiratory syncytial virus, RSV, vaccination, RSV vaccine, RSVPreF3 OA, older adults, elderly, attitudes, survey

## Abstract

*Background and Objectives*: Three respiratory syncytial virus (RSV) vaccines have been recently made available for older adults. Understanding the principal characteristics of the first vaccine-takers can pave the way for a successful vaccination campaign. The objective of this study was to explore the sociodemographic and clinical characteristics of the first Italian users of an adjuvanted RSV vaccine and their attitudes towards RSV and vaccination. *Materials and Methods*: This cross-sectional study was conducted in 2024 in Liguria (Italy). Individuals aged ≥60 years with no contraindications to the adjuvanted vaccine RSVPreF3 OA were eligible. Following vaccination, subjects filled in a questionnaire, which comprised items on sociodemographic and clinical characteristics, attitudes towards RSV and RSV vaccination and a vaccination trust indicator (VTI). *Results*: A total of 453 vaccinees completed the survey. Their mean age was 74.9 ± 8.0 years, and 50.6% were males. Nine of ten (89.2%) individuals had ≥1 co-morbidity, of which cardiovascular conditions (70.4%), respiratory diseases (27.6%) and diabetes (18.5%) were the most common. Uptake of the routine vaccines was high: 91.2% and 98.7% received the 2023/2024 season influenza and ≥2 COVID-19 vaccines, respectively. The most common reasons for the current RSV vaccination were general practitioner advice (43.9%), followed by the willingness to be protected against (20.8%) and feelings of being at risk (16.6%) of RSV. The average VTI score was 91.5%, suggesting high trust in vaccines. More positive attitudes towards RSV vaccination were observed (*p* < 0.01) among subjects who received more COVID-19 vaccine doses, whose reasons for the current RSV vaccination were the willingness to be protected or to be in good health and the feeling of being at risk for RSV. *Conclusions*: The first Italian users of the novel RSVPreF3 OA vaccine were represented by high-risk individuals with a comparatively high prevalence of co-morbidities, high uptake of the seasonal respiratory vaccines and high trust in immunization.

## 1. Introduction

Respiratory syncytial virus (RSV) is a major cause of acute respiratory infections (ARIs), which sometimes lead to severe disease, complications and death [1]. The Global Burden of Disease study [2] estimated that a total of 338,495 (95% uncertainty interval: 126,555–667,109) deaths in 2019 were due to RSV. However, the burden of RSV is unevenly distributed across the population with young children and older adults being the most susceptible groups [3]. For instance, it has been estimated that the average pooled proportions for RSV incidence, hospitalization and in-hospital case fatality rates in adults aged ≥60 years are 1.62%, 0.15% and 7.13%, respectively [4].

Three vaccines for the prevention of RSV in older adults have recently become available, including the monovalent (based on RSV subtype A) recombinant RSV glycoprotein F stabilized in pre-fusion (preF) conformation vaccine adjuvanted with AS01_E_ (RSVPreF3 OA), the bivalent (based on RSV subtypes A and B) non-adjuvanted RSV preF-based vaccine (RSVpreF) and the monovalent (based on RSV subtype A) mRNA-based vaccine encoding preF (mRNA-1345) [5]. Some countries have already issued recommendations on their use and the first post-marketing vaccine effectiveness data have been recently published. For example, in the United States (US) a single dose of any approved RSV vaccine is currently recommended for all individuals aged ≥75 years, plus for adults aged 60–74 years who are at increased risk for severe RSV disease [5]. The first available post-marketing test-negative case-control study from the US [6] reported a vaccine effectiveness of 75% [95% confidence interval (CI): 50–87%) in preventing RSV-related hospitalizations in adults aged ≥60 years. This estimate is consistent with the vaccine efficacy established in phase III trials [7,8,9].

RSVPreF3 OA was the first RSV vaccine licensed in 2023 in both the US [10] and Europe [11] with the indication of prevention of lower respiratory tract disease (LRTD) caused by RSV in adults aged ≥60 years. More recently, this indication has been extended to adults at-risk aged 50–59 years [5]. In the pivotal trial of 24,966 older adults [7], RSVPreF3 OA efficacy against LRTD caused by RSV during the first season was 82.6% (96.95% CI: 57.9–94.1%). The efficacy of a single RSVPreF3 OA dose over two RSV seasons was 67.2% (97.5% CI: 48.2–80.0%) [12]. Finally, in October 2024, data on the cumulative efficacy of RSVPreF3 OA over three consecutive seasons were released [13], and the estimate was analogously high (62.9%; 97.5% CI: 46.7–74.8%).

Age is significant predictor of vaccine uptake and vaccine hesitancy [14] As vaccine acceptance differs between age classes, public health campaigns should take into account this generational difference [15,16]. A comprehensive review of factors influencing vaccine uptake by the elderly in Western society [17] identified six groups of determinants: (i) attitudes towards and beliefs about vaccination in general; (ii) perceived risk, susceptibility and severity of a vaccine-preventable disease, including personal experience; (iii) vaccine characteristics; (iv) advice and information from healthcare professionals or relatives; (v) general health-related behaviors (e.g., prior vaccinations and other preventive behaviors) and (vi) accessibility and affordability issues [17]. In the elderly, the lack of physician recommendation is the most common barrier to vaccination [18,19].

The introduction of novel vaccines may be challenging [20], and a successful immunization campaign is driven by a plethora of factors, including contextual influences (e.g., socio-cultural, the health system, economic and political factors), individual and social group influences (e.g., personal perception and peer environment) and vaccine-specific issues (i.e., related to the characteristics of a specific vaccine) [21]. In a specific case of adult RSV vaccines, individual-level determinants are of prominence. The available research suggests that laypeople’s awareness of RSV is low [22,23], and the virus is often perceived as a children’s pathogen [24]. For instance, only 32.1% of older adults in the US are aware of RSV and this proportion is significantly lower than among younger adults [22]. In parallel, knowledge of and experience with adult RSV disease among primary care physicians is insufficient [25,26]. With respect to this background, the aim of this study was to explore socio-demographic, clinical and attitudinal characteristics of the first users of the novel RSVPreF3 OA vaccine. Indeed, as exemplified by some studies on the initial rollout of COVID-19 [27] and mpox [28] vaccination campaigns, the evaluation of individual attributes associated with the early adoption of novel vaccines may help in shaping the launch of effective and targeted communication and immunization strategies.

## 2. Materials and Methods

### 2.1. Study Design and Eligibility Criteria

This cross-sectional survey was conducted in the Liguria region (northern Italy) between February and September 2024. RSVPreF3 OA (Arexvy, lot Y7H7S; GlaxoSmithKline Biologicals SA, Rixensart, Belgium) was purchased by the Department of Health Sciences, University of Genoa (Italy) directly from the manufacturer and before its availability at both public and private markets.

To enroll subjects, several recruitment strategies were used. In particular, a notice on the study and availability of a novel RSV vaccine for individuals aged ≥60 years was advertised in local newspapers, websites and on TV. Printed pamphlets were also distributed among general practitioner (GP) offices.

Potentially eligible volunteer older adults were briefed on the study aims and details and assessed for their eligibility by trained physicians. The inclusion criteria were formulated as follows: (i) age ≥ 60 years; and (ii) ability to provide informed consent. Subjects with known allergies to any ingredient of the vaccine were excluded. The RSV vaccination of individuals who had been vaccinated with any other vaccine in a preceding week was postponed by ≥1 week.

Due to the exploratory nature of this study, no hypothesis was formulated a priori. Based on previous RSV-related surveys [22,29,30], we aimed to enroll approximately 500 individuals. Indeed, for a 7-item questionnaire with an expected Cronbach’s *α* of 0.7, precision of 5% and confidence level of 95%, at least 328 subjects are needed [31].

The study protocol was approved by the Ethics Committee of Liguria Region (n. 1/2024, id 13611). All individuals provided written informed consent. The study was reported according to the STROBE (STrengthening the Reporting of OBservational studies in Epidemiology) guidelines (Supplementary Table S1) [32].

### 2.2. Study Procedures

Upon receipt of an informed consent, the study vaccine was administered intramuscularly in the deltoid region of the non-dominant arm. Subjects were then observed for 30 min for immediate allergic reactions.

During the observation period, a trained physician interviewed vaccinees to collect socio-demographic and medical history data. After that, subjects filled in a questionnaire on knowledge of and attitudes towards RSV and vaccination; assistance was provided on request. Finally, vaccinated subjects were provided with an ad-hoc questionnaire for monitoring adverse events in the following seven days after vaccination. The analysis of adverse events following immunization will be presented in a separate manuscript.

### 2.3. Study Questionnaire and Variables

Socio-demographic variables included data on sex, age, nationality (Italian or foreign-born) and education level (primary, lower secondary, upper secondary or vocational school and university degree). Medical history data considered smoking status (never smoker, ex-smoker and current smoker), height and weight to calculate the body mass index (BMI) and presence of underlying chronic diseases (cardio- and cerebrovascular, respiratory, gastrointestinal, renal and immunosuppressive diseases, including cancer, asplenia and autoimmune conditions). Additionally, the uptake of seasonal (seasons 2022/2023 and 2023/2024) influenza and COVID-19 (cumulative number of doses administered) vaccines was obtained from electronic vaccination registries. Medical history data declared by participants were cross-checked with their GPs.

Reasons for the RSV vaccination were investigated by asking an open-ended question: “*What is the main reason that led you to get vaccinated?*”. Qualitative responses to this item were examined through content analysis in order to reduce the data and identify core themes and meanings. In particular, individual narratives were first analyzed to identify principal keywords. After that, based on similarities, single responses were then grouped and organized into distinct thematic categories. This approach was judged the most suitable, as the overwhelming majority of responses were in single-phrase or even single-word formats [33]. The thematic categories were developed by one researcher (A.D.) and then reviewed by another investigator (D.P.); eventual disagreements were solved by consensus.

Attitudes towards RSV and RSV vaccination were measured through seven ad hoc 11-point (0 to 10, from strongly disagree to strongly agree) Likert-scale items (Supplementary Table S2). These attitudinal items were developed inside the research group with the aim to explore awareness, willingness to pay, perceived risk and impact of RSV in older adults. The questionnaire was piloted in a convenience sample (*n* = 8) of older adults undergoing other non-RSV routine vaccinations to verify whether the questions were clear, relevant and appropriate. Based on the feedback, some minor changes in the wording was made. All questions had semantically positive construct and good readability.

Finally, a recently developed and validated Vaccination Trust Indicator (VTI) [34] was used to quantify general attitudes towards vaccines. Briefly, VTI consists of six 11-point (0 to 10, from strongly disagree to strongly agree) Likert-scale items, and a total VTI score is obtained by averaging responses to each question and scaling the average to a 100-point scale. It was proposed that the total scores < 40, 40–70 and >70 indicate low, moderate and high trust in vaccines, respectively [34].

### 2.4. Data Analysis

Considering the exploratory nature of the study, only descriptive statistics were used. Categorical variables were expressed as percentages with 95% CIs, while the approximately normally distributed variable of age was summarized as mean with standard deviation (SD). No statistical imputation for eventual missing data was considered. Single Likert-scale items were summarized as medians with interquartile ranges (IQRs) [35]. The internal consistency of the Italian version of VTI and the ad-hoc questionnaire with seven attitudinal items was measured by means of Cronbach’s α, which was interpreted as excellent (≥0.90), good (0.80–0.89), acceptable (0.70–0.79), questionable (0.60–0.69), poor (0.50–0.59) and unacceptable (<0.50) [36]. Spearman’s *ρ* was used to calculate correlation coefficients between the VTI score and ad hoc attitudinal items.

In a post-hoc evaluation, a linear regression analysis was performed to predict attitudes towards RSV from a set of participants’ characteristics. In this analysis, the dependent variable was expressed as an average of the seven attitudinal items. Both univariable and multivariable models were computed. The best fitting multivariable model was selected by minimizing the Akaike information criterion. The percent of variance explained was quantified by means of the coefficient of determination *R*^2^. Eventual multicollinearity was verified by computing variance inflation factors.

Data were analyzed and visualized in Excel v. 2408 (Microsoft, Redmond, WA, USA) and R stats packages v. 4.1.0 (R Core Team, Vienna, Austria).

## 3. Results

### 3.1. Characteristics of the Study Population

A total of 453 vaccine doses were administered to as many subjects, and no volunteers met exclusion criteria. Their mean age was 74.9 years, and men and women were equally distributed. The overwhelming majority were Italians, and about two thirds declared to have completed upper secondary/vocational school or university courses. A total of 56.1% of adults were current or former smokers, while about one half of individuals was overweight or obese. Most older adults had at least one underlying chronic condition, of which cardiovascular, respiratory diseases and diabetes were the most common. Fifteen percent were cancer survivals, while 2.4% had malignancies at the time of the survey (Table 1).

Individuals with available immunization records (*n* = 445) showed high uptakes of seasonal influenza and COVID-19 vaccines. In particular, the 2022/2023 and 2023/2024 seasonal influenza vaccination coverage rates were 86.7% (95% CI: 83.2–89.8%) and 91.2% (95% CI: 88.2–93.7%), respectively. A total of 98.7% (95% CI: 97.1–99.5%) of adults completed the primary series of the COVID-19 vaccination and/or received booster doses, and the median number of doses received was four [IQR: 3–5] (Table 1).

### 3.2. Reasons for Being Vaccinated Against RSV

Following the content analysis of the open-ended question on the reasons for vaccination with RSVPreF3 OA, six categories of responses were identified and labeled as follows: (i) GP advice; (ii) to be (more) protected; (iii) feeling at risk; (iv) fear of the virus; (v) to be in (good) health and (vi) importance of vaccines in general (Table 2). GP advice was by far the most frequently (43.9%) cited reason for vaccination. Statements describing the willingness to be (more) protected and feeling of being at risk of RSV disease because of the presence of certain health conditions were reported by 20.8% and 16.6% of adults, respectively. The remaining reasons were reported by less than 5% of respondents.

### 3.3. Attitudes Towards RSV and RSV Vaccines and Trust in Vaccination

**Figure 1:**
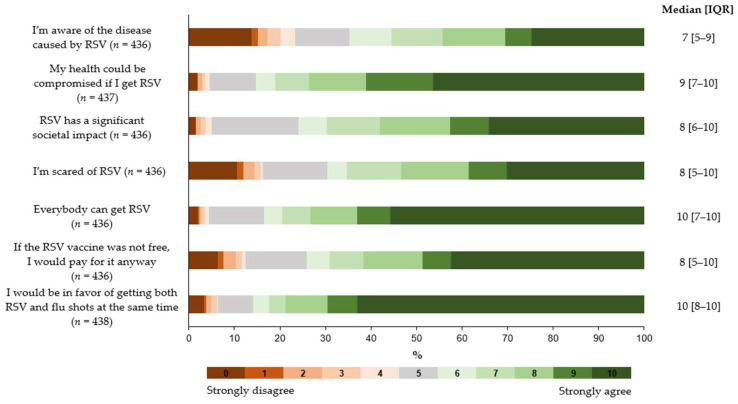
Attitudes towards RSV and RSV vaccination.

The questionnaire on the attitudinal items and VTI was completed by 438 individuals (96.7%). The seven-item attitudinal questionnaire showed an acceptable internal consistency with a Cronbach *α* of 0.74 (95% CI: 0.70–0.78). As shown in Figure 1, most participants agreed to some degree with the statements of all attitudinal items. The highest agreement (median scores of 10) was observed for the items on the possibility of RSV and influenza vaccines co-administration and on the fact that everyone can be infected with RSV. On the other hand, about one fourth of subjects indicated low awareness of RSV disease.

Results of the post-hoc regression analysis to predict the average score of seven attitudinal factors are reported in Table 3. In the adjusted analysis, more positive attitudes towards RSV and RSV vaccination were associated with a greater cumulative number of COVID-19 vaccine doses received (*p* = 0.005). Compared with individuals whose main reason for the current RSV vaccination was GP advice, those whose reasons were the willingness to be protected (*p* < 0.001) or to be in good health (*p* = 0.002) and feeling of being at risk for RSV (*p* = 0.004) were associated with more positive attitudes. Completion of university studies was significant only in the unadjusted model. However, both multivariable models explained only about 11% of variance.

The Cronbach *α* of the VTI was deemed good (0.87; 95% CI: 0.85–0.89). The average VTI score was 91.5 (SD 11.2) points, and 0.7% (95% CI: 0.1–2.0%), 3.9% (95% CI: 2.3–6.1%) and 95.4% (95% CI: 93.0–97.2%) of respondents had low, moderate and high trust in vaccines, respectively. The total VTI score correlated significantly (*p* < 0.01) with all seven attitudinal items with correlation coefficients ranging from 0.16 to 0.41 (Supplementary Table S3).

## 4. Discussion

This study is the first to explore the characteristics of a cohort of Italian older adults immunized with an RSV vaccine, which is currently not included in the National Immunization Schedule. Compared with the general population of older adults, the first takers of the novel RSV vaccine had more co-morbidities and had a comparatively high uptake of other respiratory vaccines. It then emerged that the main reason for RSV vaccination was GP advice, followed by the willingness to be protected from the virus and feeling of being at risk of the disease. We finally showed that the first RSV vaccine users declared generally positive attitudes towards RSV vaccines, and their trust in vaccination was comparatively high. More positive attitudes towards RSV vaccination may be associated with both a greater perceived risk of RSV and greater gains in health. Taken together, we believe that these preliminary findings may support local decision makers in the implementation of the future RSV vaccination campaign.

In this study, the overwhelming majority (89.2%) of older adults had at least one co-morbidity, which is significantly higher than the regional estimate of 54.9% [37]. This difference was primarily driven by the presence of chronic respiratory and cardiovascular conditions. The presence of co-morbidities and, in particular, an increased number of chronic conditions is a well-established positive predictor of influenza [38,39], pneumococcal [40] and COVID-19 [41] vaccinations. Some previous research highlighted that the presence of underlying diseases may enhance risk perception of respiratory viruses. In particular, in a recent US survey of a nationally representative sample of adults [42], compared with immunocompetent individuals, those who were immunocompromised were about twice as many times more worried about becoming seriously ill from RSV (24% vs. 43%).

As expected, seasonal influenza and COVID-19 vaccine uptake in our study was significantly higher than the regional estimates. For example, regional influenza vaccination coverage rates in adults aged ≥65 years were 53.7% for the season 2022/2023 and 54.5% for the season 2023/2024 [43], while, in this study, the corresponding uptake rates were 86.7% and 91.2%, respectively. This difference is likely driven by both a higher prevalence of co-morbidities and high confidence in vaccination declared by individuals in our study. Acceptance of different respiratory vaccines may be interrelated [44]. Indeed, previous influenza vaccination is a well-known positive predictor of COVID-19 vaccination [45,46], and, in this study, more positive attitudes towards RSV and RSV vaccination was positively associated with the total number of COVID-19 vaccine doses received. We, therefore, believe that prior experience with other respiratory vaccines (in particular, seasonal influenza and COVID-19) should be leveraged to promote RSV vaccination. Luisi et al. [47] suggest that healthcare providers should use vaccination appointments to promote other immunizations. In turn, counselling interventions among individuals seeking for one vaccine may improve the uptake of other recommended vaccines [48].

About 80% of older adults were in favor of influenza and RSV vaccines co-administration. Randomized controlled trials (RCTs) showed [49,50,51] that the available RSV and seasonal influenza vaccines may be co-administered with no clinically significant interference in terms of safety and immunogenicity towards antigens contained in both vaccines. The simultaneous administration of two or more vaccines during the same visit has a number of advantages, including a reduction in the number of visits and related costs, improvement in compliance with official recommendations and insurance of the timely vaccine administration according to the recommended schedule, especially in the case of newly licensed vaccines [52]. It should be, however, stressed that the high acceptance of vaccine co-administration may not lead to high co-administration rates. For instance, in a nationally representative Italian study [53], only 16.6% of adults declared their firm unwillingness to receive COVID-19 and influenza vaccines simultaneously. On the other hand, despite the availability of several RCTs [54] and national recommendations on the co-administration of COVID-19 and influenza vaccines [55], the effective rate of co-administration may be as low as 11% [56].

When scrutinizing reasons for the RSV vaccination, GP advice was the most frequently reported motivational factor. GPs will therefore play the primary role in promoting RSV vaccination among their patients. The pivotal role of GPs in recommending influenza vaccination is a well-known independent correlate associated with the actual vaccine receipt [40,57]. This recommendation can also compensate for the effects of hesitancy associated with novel vaccines [58], such as RSV vaccines. Considering that a significant proportion of GPs may have insufficient knowledge of RSV disease and prevention in (older) adults [25,26], targeted interventions to increases GPs’ awareness of the topic should be established by local health authorities, scientific societies and other institutional stakeholders before the implementation of a vaccination campaign. In this regard, de Lusignan et al. [59] evaluated changes in knowledge, attitudes and behaviors of GPs and their patients before and after attendance of a continuing medical education (CME) program during the 2018/2019 influenza season in the United Kingdom. The CME course was established in order to educate and familiarize GPs with a newly available adjuvanted influenza vaccine, preferentially recommended to older adults. It emerged that the CME module increased GP confidence in the ability to address people’s concerns about influenza and vaccination. Analogously, after meeting their GPs, patients reported significantly higher confidence in the effectiveness and safety of the adjuvanted influenza vaccine. In this study, influenza vaccination coverage was 10% higher than the English national average (82.2% vs. 72.0%) [59].

We than found that individuals whose main reasons for the current RSV vaccination were related to personal risk perception and overall well-being (as opposed to GP recommendation) gave higher rankings to the items on the attitudes towards RSV and RSV vaccination. Alfano and Ercolano [60] identified two channels through which an individual takes a positive attitude to vaccination, namely individualistic and altruistic. The former includes reasons linked to individual gains associated with immunization (e.g., reduced risk of infection), while the latter reasons are related to the collective community benefits due to vaccination. Our findings suggest that the individualistic dimension of RSV vaccination attitudes may play an important role in future communication campaigns. Communication campaigns developed to change risk perceptions are effective to increase vaccination uptake. Betsch et al. [61] suggest that fear appeals can increase the perceived risk of vaccine-preventable diseases. In this regard, an Italian study [28] documented that risk awareness is a major determinant of the early acceptance of a novel mpox vaccine.

The major limitation of this study concerns the inclusion of only vaccinees, and, as such, our results may lack generalizability if compared with future population-level studies (i.e., those studies enrolling individuals independently from their RSV vaccination status). As also discussed earlier, the inclusion of only adults vaccinated against RSV determined a higher-than-average prevalence of co-morbidities, the uptake of other respiratory vaccines and likely better confidence in vaccination. Future population-level surveys are warranted to shed light on the determinants of RSV uptake. Furthermore, although the seven-item attitudinal survey instrument showed an acceptable level of internal consistency and correlated with the total VTI score, this tool had not been fully validated, and other types of internal and external validities had not been tested. Finally, this ancillary study investigated only a limited number of factors associated with actual RSV vaccine receipt and attitudes towards RSV and RSV vaccination. Future studies should scrutinize a more comprehensive set of enablers and barriers, as, for example, described by Eilers and colleagues [17].

In conclusion, our preliminary findings suggest that the initial pool of RSV vaccine takers will be likely composed of high-risk individuals with a comparatively high prevalence of cardiovascular and respiratory diseases, who have high uptake of other seasonal vaccines and who show high confidence in vaccines. GPs will play a pivotal role in promoting RSV vaccine receipt. Future communication and promotion campaigns should not only aim at increasing general RSV awareness, but also at enhancing positive attitudes towards vaccination through reinforcement of personal risk perception and health gains. Offering RSV vaccination during appointments for other routine vaccinations, such as COVID-19 or seasonal influenza, seems promising.

## Figures and Tables

**Table 1:** Characteristics of the older adults vaccinated with RSVPreF3 OA (*n* = 453).

Variable	Level	*n* (%)
Sex	Male	229 (50.6)
	Female	224 (49.4)
Age, years	Mean (SD)	74.9 (8.0)
	60–74	220 (48.6)
	≥75	233 (51.4)
Nationality	Italian	440 (97.1)
	Foreign-born	13 (2.9)
Education level	Primary school	58 (12.8)
	Lower secondary school	84 (18.5)
	Upper secondary/vocational school	175 (38.6)
	University	128 (28.3)
	Not available	8 (1.8)
Smoking habits	Never smoker	199 (43.9)
	Ex-smoker	205 (45.3)
	Current smoker	49 (10.8)
Body mass index, kg/m^2^	<18.5	14 (3.1)
	18.5–24.9	221 (48.8)
	25.0–29.9	158 (34.9)
	≥30.0	60 (13.2)
Presence of co-morbidities	≥1	404 (89.2)
	Cardiovascular (incl. hypertension)	319 (70.4)
	Cardiovascular (excl. hypertension)	160 (35.3)
	Cerebrovascular	46 (10.2)
	Respiratory	125 (27.6)
	Diabetes	84 (18.5)
	Gastrointestinal	41 (9.1)
	Renal	23 (5.1)
	Autoimmune/rheumatic	29 (6.4)
	Asplenia	3 (0.7)
	Current cancer	11 (2.4)
	Past cancer	68 (15.0)
2022/2023 season influenza vaccination	Yes	386 (85.2)
	No	59 (13.0)
	Not available	8 (1.8)
2023/2024 season influenza vaccination	Yes	406 (89.6)
	No	39 (8.6)
	Not available	8 (1.8)
COVID-19 vaccination, cumulative number of doses	0	6 (1.3)
	1	0 (0)
	2	10 (2.2)
	3	108 (23.8)
	4	118 (26.0)
	5	158 (34.9)
	6	45 (9.9)
	Not available	8 (1.8)

**Table 2:** Main reasons for being vaccinated against respiratory syncytial virus (*n* = 453).

Reason	*n* (%)	95% CI
General practitioner advice	199 (43.9)	39.3–48.6
To be (more) protected	94 (20.8)	17.1–24.8
Feeling at risk	75 (16.6)	13.3–20.3
Fear of the virus	17 (3.8)	2.2–5.9
To be in (good) health	16 (3.5)	2.0–5.7
Importance of vaccines in general	9 (2.0)	0.9–3.7
Other	22 (4.9)	3.1–7.3
Not available	21 (4.6)	–

CI, confidence interval.

**Table 3:** Linear regression models to predict attitudes towards respiratory syncytial virus.

Variable	Level	*b* (SE) ^1^	*b* (SE) ^2^	*b* (SE) ^3^
Sex	Male	Reference	–	Reference
	Female	0.177 (0.164)	–	0.121 (0.163)
Age, years	1-year increase	−0.001 (0.010)	–	−0.003 (0.012)
Nationality	Italian	Reference	–	Reference
	Foreign-born	0.221 (0.484)	–	0.151 (0.477)
Education level	Primary school	Reference	Reference	Reference
	Lower secondary school	−0.087 (0.294)	−0.353 (0.292)	−0.358 (0.301)
	Upper secondary/vocational school	0.034 (0.261)	−0.271 (0.263)	−0.274 (0.280)
	University	0.633 (0.273) *	0.203 (0.281) *	0.189 (0.300)
Presence of co-morbidities	No	Reference	–	Reference
	Yes	−0.059 (0.263)	–	0.039 (0.274)
2022/2023 season influenza vaccination	No	Reference	–	Reference
	Yes	0.097 (0.242)	–	−0.060 (0.264)
2023/2024 season influenza vaccination	No	Reference	–	Reference
	Yes	0.223 (0.305)	–	0.226 (0.327)
COVID-19 vaccination	Each additional dose	0.247 (0.072) ***	0.209 (0.074) **	0.205 (0.082) *
Reasons for vaccination against respiratory syncytial virus	General practitioner advice	Reference	Reference	Reference
	To be (more) protected	0.938 (0.208) ***	0.789 (0.210) ***	0.772 (0.218) ***
	Feeling at risk	0.865 (0.225) ***	0.681 (0.232) **	0.686 (0.239) **
	Fear of the virus	0.841 (0.420) *	0.573 (0.421)	0.579 (0.426)
	To be in (good) health	1.415 (0.432) **	1.387 (0.443) **	1.402 (0.450) **
	Importance of vaccines in general	−0.431 (0.566)	−0.729 (0.567)	−0.717 (0.575)
	Other	0.860 (0.373) *	0.828 (0.379) *	0.839 (0.385) *

^1^ Unadjusted coefficients estimated through univariable models; ^2^ Adjusted coefficients estimated through a multivariable model selected by minimizing the Akaike information criterion; ^3^ Adjusted coefficients estimated through a full multivariable model; *** *p* < 0.001; ** *p* < 0.01; * *p* < 0.05. SE, standard error.

## Data Availability

All relevant data of this descriptive study are within the manuscript and associated Supplementary Materials. Further details may be available by the authors on request.

## Supplementary Materials

The following supporting information can be downloaded at: https://www.mdpi.com/article/10.3390/medicina61010067/s1, Table S1: STROBE (STrengthening the Reporting of OBservational studies in Epidemiology) checklist; Table S2: Likert scale-based survey items on the attitudes towards respiratory syncytial virus (RSV) and RSV vaccination; and Table S3: Spearman’s *ρ* correlation coefficients between seven attitudinal Likert scale-based items and the total vaccination trust indicator (VTI) score.
