# Supplementary material for: Characteristics of the First Italian Older Adults Vaccinated with an Adjuvanted Respiratory Syncytial Virus (RSV) Vaccine
Source: Medicina (Kaunas). 2025 Jan 3;61(1):67. doi: 10.3390/medicina61010067 (PMC11766504; doi:10.3390/medicina61010067)
Supplement: Supplementary file 1 [file medicina-61-00067-s001.zip › medicina-3396114-supplementary.pdf]

Table S1. STROBE (STrengthening the Reporting of OBservational studies in Epidemiology) checklist.

	Item No	Recommendation	Reported in section (§)
**Title and abstract**	1	(*a*) Indicate the study's design with a commonly used term in the title or the abstract	Abstract
		(*b*) Provide in the abstract an informative and balanced summary of what was done and what was found	Abstract
**Introduction**			
Background/rationale	2	Explain the scientific background and rationale for the investigation being reported	1 (1–5)
Objectives	3	State specific objectives, including any prespecified hypotheses	1 (5)
**Methods**			
Study design	4	Present key elements of study design early in the paper	2.1 (1)
Setting	5	Describe the setting, locations, and relevant dates, including periods of recruitment, exposure, follow-up, and data collection	2.1 (1, 2)
Participants	6	(*a*) Give the eligibility criteria, and the sources and methods of selection of participants	2.1 (2, 3)
Variables	7	Clearly define all outcomes, exposures, predictors, potential confounders, and effect modifiers. Give diagnostic criteria, if applicable	2.3 (1–4)
Data sources/ measurement	8	For each variable of interest, give sources of data and details of methods of assessment (measurement). Describe comparability of assessment methods if there is more than one group	2.3 (1–4)
Bias	9	Describe any efforts to address potential sources of bias	2.2 (2)
Study size	10	Explain how the study size was arrived at	2.1 (4)
Quantitative variables	11	Explain how quantitative variables were handled in the analyses. If applicable, describe which groupings were chosen and why	2.3 (3,4); 2.4 (1)
Statistical methods	12	(*a*) Describe all statistical methods, including those used to control for confounding	2.4 (1, 2)
		(*b*) Describe any methods used to examine subgroups and interactions	NA
		(*c*) Explain how missing data were addressed	2.4 (1)
		(*d*) If applicable, describe analytical methods taking account of sampling strategy	NA
		(*e*) Describe any sensitivity analyses	NA

**Results**			
Participants	13	(a) Report numbers of individuals at each stage of study—e.g. numbers potentially eligible, examined for eligibility, confirmed eligible, included in the study, completing follow-up, and analyzed	3 (1)
		(b) Give reasons for non-participation at each stage	NA
		(c) Consider use of a flow diagram	NA
Descriptive data	14	(a) Give characteristics of study participants (e.g. demographic, clinical, social) and information on exposures and potential confounders	3.1 (Table 1)
		(b) Indicate number of participants with missing data for each variable of interest	3.1 (Table 1)
Outcome data	15	Report numbers of outcome events or summary measures	3.1 (Table 1); 3.2 (Table 2); 3.3 (Figure 1; Table 3)
Main results	16	(*a*) Give unadjusted estimates and, if applicable, confounder-adjusted estimates and their precision (e.g., 95% confidence interval). Make clear which confounders were adjusted for and why they were included	3.1 (Table 1); 3.2 (Table 2); 3.3 (1, Figure 1)
		(*b*) Report category boundaries when continuous variables were categorized	NA
		(*c*) If relevant, consider translating estimates of relative risk into absolute risk for a meaningful time period	NA
Other analyses	17	Report other analyses done—e.g. analyses of subgroups and interactions, and sensitivity analyses	NA
**Discussion**			
Key results	18	Summaries key results with reference to study objectives	4 (1)
Limitations	19	Discuss limitations of the study, taking into account sources of potential bias or imprecision. Discuss both direction and magnitude of any potential bias	4 (7)
Interpretation	20	Give a cautious overall interpretation of results considering objectives, limitations, multiplicity of analyses, results from similar studies, and other relevant evidence	4 (2–6)
Generalizability	21	Discuss the generalizability (external validity) of the study results	4 (7)
**Other information**			
Funding	22	Give the source of funding and the role of the funders for the present study and, if applicable, for the original study on which the present article is based	Funding

NA, Not applicable.

Table S2. Likert scale-based survey items on the attitudes towards respiratory syncytial virus (RSV) and RSV vaccination.

#	Item
1	I'm aware of the disease caused by RSV
2	My health could be compromised if I get RSV
3	RSV has a significant society impact
4	I'm scared of RSV
5	Everybody can get RSV
6	If the RSV vaccine was not free, I would pay for it anyway
7	I would be in favor of getting both RSV and flu shots at the same time

Note: For each item, the level of agreement was measured on an 11-point scale ranging from 0 to 10 (where 0 = strongly disagree and 10 = strongly agree).

Table S3. Spearman's ρ correlation coefficients between seven attitudinal Likert scale-based items and the total vaccination trust indicator (VTI) score.

#	Item	ρ	95% CI
1	I'm aware of the disease caused by RSV	0.21	0.12–0.31
2	My health could be compromised if I get RSV	0.38	0.29–0.46
3	RSV has a significant society impact	0.34	0.26–0.43
4	I'm scared of RSV	0.16	0.07–0.26
5	Everybody can get RSV	0.26	0.17–0.35
6	If the RSV vaccine was not free, I would pay for it anyway	0.41	0.32–0.49
7	I would be in favor of getting both RSV and flu shots at the same time	0.40	0.31–0.48

Note: For each item, the level of agreement was measured on an 11-point scale ranging from 0 to 10 (where 0 = strongly disagree and 10 = strongly agree).
CI, confidence interval.
